# P-1200. Real-World Utilization of Rezafungin in the Outpatient Setting

**DOI:** 10.1093/ofid/ofaf695.1393

**Published:** 2026-01-11

**Authors:** Nicholas Van Hise, Mark A Redell, Jalal A Aram, Vishnu Chundi, Ingrid Roig, Bala Somayaji, Samir Allos, Jaime Belmares, Michael Anderson, Robert Fliegelman, Russell M Petrak

**Affiliations:** MIDC, Burr Ridge, Illinois; Melinta Therapeutics, Parsippany, NewJersey; Melinta Therapeutics, Parsippany, NewJersey; Metro Infectious Disease Consultants, Chicago, Illinois; Metro Infectious Disease Consultants, Chicago, Illinois; Metro Infectious Disease Consultants, Chicago, Illinois; Metro Infectious Disease Consultant, Huntsville, Alabama; Metro Infectious Disease Consultants, Chicago, Illinois; Metro Infectious Disease Consultants, Chicago, Illinois; Metro Infectious Disease Consultants, Chicago, Illinois; Metro Infectious Disease Consultants, Chicago, Illinois

## Abstract

**Background:**

Rezafungin (RZF), a novel intravenous echinocandin with an extended half-life of 152 hours, was approved in the US in 2023 for adult patients with candidemia or invasive candidiasis as a once weekly dosing regimen. This provides advantages in various healthcare settings by improving compliance, removing the risks associated the use of long term central venous catheters and daily antifungal administration. This study describes our real-world experience with RZF in an outpatient setting.

**Methods:**

A retrospective cohort study was conducted across 23 treatment sites, with 75 providers treating 86 total patients. Forty-seven patients were transitioned from inpatient to outpatient utilizing RZF. All patients from May 2024 to December 2024 that received ≥ 1 dose of RZF were evaluated. Data collection included demographics, medical history, treatment details, microbiology, and safety/tolerability. Primary outcomes of infection and safety were assessed by the treating provider.. All cause adverse events were reported and evaluated for relation to RZF.

**Results:**

The average (avg) age was 62 years of age (23-91), avg weight 85.2 kg (47kg-187kg) and avg CrCL was 77.4 mL/min; 8 patients had CKD. Patients received an average of 3.58 doses of RZF. Primary clinical outcomes: 57 (66%) resolved, 18 (21%) improved, and 11 (13%) were unsuccessful. In addition, 11 patients experienced an adverse event; four of these reactions were determined to be related to RZF. One adverse event was determined to be serious. Forty patients were determined to have invasive candidiasis or candidemia. Other diagnoses ranged as follows: esophageal candidiasis (23), vulvovaginal candidiasis (14), and others.

**Conclusion:**

Rezafungin was a safe and effective treatment for candidemia and invasive candidiasis and other infection types. Additionally, this retrospective study provides real-world evidence and support for the use of RZF as a potentially important agent in the outpatient setting.

**Disclosures:**

Nicholas Van Hise, PharmD, Melinta Therapeutics: Grant/Research Support Mark A. Redell, PharmD, Melinta Therapeutics: Full-time employee Jalal A. Aram, MD, Melinta Theraputics: Employee

